# *Salvia rosmarinus* Antinociceptive Efficacy Depends on Sex and Involves Hormonal Mechanisms in Experimental Fibromyalgia-Like Pain

**DOI:** 10.3390/ijms27198580

**Published:** 2026-09-25

**Authors:** Janet Siles-Guevara, María Eva González-Trujano, Erika M. Estrada-Camarena, Berenice Ovalle-Magallanes, Hermelinda Salgado-Ceballos, Lucía A. Martínez-Mota, Myrna Déciga-Campos

**Affiliations:** 1 Laboratorio de Neurofarmacología de Productos Naturales, Dirección de Investigaciones Biomédicas en Salud Mental, Instituto Nacional de Psiquiatría Ramón de la Fuente Muñiz, Calz. México-Xochimilco 101, Col. San Lorenzo Huipulco, Tlalpan, Ciudad de México 14370, Mexico; jsilesg2000@alumno.ipn.mx; 2 Sección de Estudios de Posgrado e Investigación de la Escuela Superior de Medicina, Instituto Politécnico Nacional, Plan de San Luis y Díaz Mirón s/n, Col. Casco de Santo Tomas, Alcaldía Miguel Hidalgo, Ciudad de México 11340, Mexico; 3 Laboratorio de Neuropsicofarmacología, Dirección de Investigaciones Biomédicas en Salud Mental, Instituto Nacional de Psiquiatría Ramón de la Fuente Muñiz, Calz. México-Xochimilco 101, Col. San Lorenzo Huipulco, Tlalpan, Ciudad de México 14370, Mexico; monesca71@gmail.com or estrada@inprf.gob.mx; 4 Departamento de Farmacia, Facultad de Química, Universidad Nacional Autónoma de México, Ciudad de México 04510, Mexico; ovalle.magallanes@quimica.unam.mx; 5 Unidad de Investigación Médica en Enfermedades Neurológicas, Hospital de Especialidades Dr. Bernardo Sepúlveda, Centro Médico Nacional Siglo XXI, Instituto Mexicano del Seguro Social, Ciudad de México 06720, Mexico; melisalce@yahoo.com; 6 Subdirección de Investigaciones Clínicas, Instituto Nacional de Psiquiatría Ramón de la Fuente Muñiz, Calz. México-Xochimilco 101, Col. San Lorenzo Huipulco, Tlalpan, Ciudad de México 14370, Mexico; lucia@inprf.gob.mx

**Keywords:** allodynia, hyperalgesia, fibromyalgia, medicinal plants, nociplastic pain, *Salvia rosmarinus*, sex

## Abstract

Fibromyalgia (FM) is a chronic, widespread musculoskeletal pain syndrome of still unknown etiology, characterized by allodynia and hyperalgesia. It affects between 2% and 8% of the population and is significantly more prevalent in women than in men. It is attributed to physiological, endocrine, immunological, and neurobiological variations that influence both pain development and the response to pharmacological treatments. Limited efficacy and a high incidence of adverse effects of current pharmacological treatments for FM have increased interest in the use of alternative therapies with medicinal plants. Non-polar extracts such as the essential oil of *Salvia rosmarinus* are included in herbal formulations, suggesting their potential properties against nociplastic pain. However, the evidence regarding the efficacy and mechanisms of action according to sex remains unexplored. The objective in this study was to investigate the efficacy of a non-polar extract of *S. rosmarinus*, whose GC-MS profile is described, and to explore the involvement of gonadal hormone receptors in an experimental model of FM-like pain in male and female rats. A hexane extract of the aerial parts of *S. rosmarinus* (10, 30 and 300 mg/kg, i.p.) or gabapentin (30 mg/kg, i.p., as a reference drug) was evaluated 30 min after its administration by using the Von Frey filaments and thermal acetone test for tactile and mechanical allodynia, respectively, and the Randall Sellito test for mechanical hyperalgesia was carried out for a period of 240 min. To determine the involvement of gonadal hormone receptors, reserpinized female rats were pretreated with the selective estrogen receptor β antagonist (ERβ, PHTPP, 25 µg/rat) 24 h before the extract, while male rats received non-steroidal antiandrogen pretreatment with flutamide (10 mg/kg/day) for 14 days, followed by behavioral testing. The extract produced a dose-dependent antiallodynic and antihyperalgesic effects, with greater efficacy in females compared to males, as the effects were significant starting at a dose of 30 mg/kg, while in males only the highest dose was significant. Blockade of ERβ receptors inhibited the extract effects in females, while androgen blockade facilitated the effects in males. The results of this study suggest that non-polar metabolites are involved in the antiallodynic and antihyperalgesic efficacy of *S. rosmarinus* mediated by sex-dependent gonadal hormonal mechanisms for FM-type pain relief.

## 1. Introduction

Fibromyalgia (FM) is a chronic and generalized musculoskeletal pain syndrome of still unknown etiology, characterized by the presence of allodynia, hyperalgesia, and spontaneous pain, and accompanied by systemic symptoms such as fatigue, headache, gastrointestinal disorders, cognitive dysfunction, anxiety, and depression, which significantly impair the quality of life of patients [1]. It is considered one of the most prevalent musculoskeletal diseases in the world [2], affecting approximately 2 to 5% of the population that can strike at any age and ethnic group, which might worsen with determined lifestyle characteristics [3]. FM has a marked adult female predominance, as 80% and 96% of diagnosed patients are women [4], but also adult men and children are among the patients who suffer from this debilitating disease [5]. This disorder is often seen in families, among siblings or mothers and their children [6].

A higher prevalence in women is generally observed in chronic pain. Although historically this difference was mainly attributed to psychosocial factors, there is currently growing evidence that sex-dependent biological mechanisms play an important role in pain susceptibility and expression [7]. Several studies have shown that males and females differ in physiological, endocrine, immunological, and neurobiological aspects that can influence both pain development and the response to pharmacological treatments [7,8]. Although most patients with chronic pain are women, it is a fact that much of the preclinical research continues to be conducted in male animals [7]. Consequently, international organizations such as the National Institutes of Health have promoted the inclusion of both sexes in preclinical research to improve the reproducibility and translation of findings [2,8].

The pathophysiology of FM is not yet fully understood, which complicates both its diagnosis and the development of effective therapeutic strategies, as well as understanding the differences between men and women [9]. Hormones can modify the response to analgesic treatments, contributing to differences observed between males and females in both experimental models and clinical conditions of chronic pain [10]. Thus, from an endocrine perspective, estrogens and androgens have been shown to modulate nociceptive sensitivity through their effects on sensory neurons, central circuits, and the immune system [11]. Estrogens participate in the regulation of multiple pathways involved in pain transmission, while testosterone appears to exert predominantly antinociceptive effects [12]. Currently, pharmacological treatment is used in both women and men, focusing on symptom relief and primarily employing central nervous system (CNS) modulators, such as the antidepressants duloxetine and milnacipran, as well as the anticonvulsants pregabalin and gabapentin, among others [13,14]. However, these treatments have limited efficacy and are often associated with adverse effects [15].

Given the limitations of conventional treatments, interest in alternative therapies based on natural products has increased, as is the case with *Salvia rosmarinus* Spenn. (formerly known as *Rosmarinus officinalis* L.), a medicinal plant species with potential for pain management due to its wide spectrum of analgesic activity supported by antinociceptive and anti-inflammatory activities but also a potent antioxidant property in different kinds of pain [16]. Regarding its preparation, the essential oil of *S. rosmarinus* has been prescribed in humans topically for muscular and rheumatic pain, as well as trauma [17]. Its antinociceptive and anti-inflammatory properties have been reported in experimental models performed in male rats and mice, such as after oral administration of the oil (70–750 mg/kg), to significantly reduce carrageenan-induced edema, pleural effusion volume, and acetic acid-induced abdominal contortions, but did not alter the response in the hot plate test, suggesting that its analgesic effects are primarily related to peripheral mechanisms associated with inflammation [18]. Nevertheless, the essential oil also showed a dose-dependent antinociceptive effect (100–600 mg/kg, i.p.) in a model of arthritic pain in male rats, where the blockage of the 5-HT_1A_ serotonin and endogenous opioid receptors significantly reduced this effect, supporting the involvement of serotonergic and endogenous opioid mechanisms in the analgesic activity due not only to peripheral but also possible CNS activity [19]. A similar non-polar preparation of *S. rosmarinus* is obtained by extracting with hexane, which compounds share similarities with bioactive molecules capable of interacting with hormonal signaling systems [20]. Some of its components, such as phenolic diterpenes like carnosol and carnosic acid [21], have been shown to interact with estrogen and androgen receptors, suggesting a possible modulatory effect on sex-dependent hormonal pathways [20]. However, whether *S. rosmarinus* extract can reduce chronic pain such as FM and whether its effects differ between sexes is unknown.

The reserpine-induced FM-like pain model was selected because it produces persistent nociceptive alterations, such as allodynia and hyperalgesia, relevant pain characteristics observed in human FM. The use of male and female Wistar rats allowed us to identify that the treatment’s efficacy on nociceptive responses may be sex dependent, partly due to hormonal influence, which is particularly relevant considering the marked prevalence of FM in women compared to men. The interest of this investigation is to look for the antihiperalgesic and antiallodynic-like responses of a non-polar extract of this medicinal plant to discriminate if its efficacy is dependent on sex by evaluating female and male Wistar rats in the reserpine-induced FM-like pain and by identifying the involvement of estrogen and/or androgen receptors, respectively.

## 2. Results

### 2.1. Gas Chromatography Coupled to Mass Spectrometry (GC-MS) of S. rosmarinus Hexane Extract

GC-MS analysis identified 44 peaks representing the total chromatogram area of the hexane extract of *S. rosmarinus* (Figure 1). Sixteen of these peaks (83.18% of the total area) corresponded to natural products (Figure 2); the remainder (0.26%) corresponded to column bleed, to unknown compounds, or to compounds with no matches (16.56%) in the NIST library. (+)-2-bornanone (camphor, 24.47%) was the main component of this extract. The identified natural products are listed in Table 1.

### 2.2. Effects of the Hexane Extract of the Aerial Parts of S. rosmarinus on Reserpine-Induced Tactile Allodynia in Female and Male Rats

*S. rosmarinus* extract produced an anti-allodynic effect in both male and female rats. But in female rats, the effect was significant from 10 mg/kg observed up to 210 and 240 min after administration (Figure 3A). This effect was enhanced after a dosage of 30 mg/kg, at which significance was observed at 30 min, with a maximum effect reached at 90 min that persisted until the end of the evaluation period. This response was comparable to that observed with GBP at the same dose, but the reference drug was significant until 120 min. The effect of the extract was improved when the dose was increased to 300 mg/kg, but it started at 60 min and reached its maximum at 210 min (Treatment: F_5,270_ = 118.80, *p* < 0.0001; Time: F_8,270_ = 17.57, *p* < 0.0001; Interaction: F_40,270_ = 3.00, *p* < 0.0001) (Figure 3A).

In male rats, the extract at 10 and 30 mg/kg did not produce significant effects. While an antiallodynic response was significantly obtained when the dose was increased to 300 mg/kg, starting with the maximum effect at 60 min and remaining until the end of the evaluation period. This effect was equivalent to that obtained with GBP (30 mg/kg, i.p.), which produced a significant antiallodynic effect after 120 min of the administration, with a maximal effect observed at 240 min (Treatment: F_5,270_ = 97.94, *p* < 0.0001; Time: F_8,270_ = 3.97, *p* = 0.0002; Interaction: F_40,270_ = 1.584, *p* = 0.0185) (Figure 3B).

The AUC_0–240 min_ from the time course curves showed that the withdrawal threshold was significantly and similarly reduced in reserpinized female and male rats (Figure 3C). In all rats, the 10 mg/kg extract showed the lowest antiallodynic response (14.94 ± 0.20% and 18.19 ± 2.02%, respectively), which improved in female rats (74.93 ± 3.58%) but not in male rats (18.34 ± 2.04%) when the dose was increased to 30 mg/kg. The significant effect was maintained in female rats when the extract dose was increased to 300 mg/kg (46.34 ± 1.73%), whereas significance was reached in male rats only at this dose (69.27 ± 10.29%) (Treatment: F_5,60_ = 45.96, *p* < 0.0001; Sex: F_1,60_ = 0.1981, *p* = 0.6578; Interaction: F_5,60_ = 6.912, *p* < 0.0001) (Figure 3C). A greater anti-allodynic response was observed in females (74.93 ± 3.58%) compared to the group treated with GBP (46.40 ± 3.69%) (t_9.572_ = 3.351, *p* = 0.0078) at the same dose of 30 mg/kg (Figure 3C).

### 2.3. Effect of the Hexane Extract of the Aerial Parts S. rosmarinus Extract on Reserpine-Induced Thermal Allodynia in Female and Male Rats

In contrast to tactile allodynia, *S. rosmarinus* extract produced a significant effect in thermal allodynia equivalent in both male and female rats. In addition, female rats showed a similar anti-allodynic response in all three doses evaluated (Figure 4A). This effect was significant from the first 30 min after administration; the effect reached a stable maximum intensity at approximately 120 min remaining until the end of the evaluation period. The effect was comparable to that observed with GBP (30 mg/kg) (Treatment: F_5,270_ = 83.98, *p* < 0.0001; Time: F_8,270_ = 12.48, *p* < 0.0001; Interaction: F_40,270_ = 2.27, *p* < 0.0001) (Figure 4A).

In male rats as in female rats, the antiallodynic response was equivalent in all three doses evaluated (Figure 4B). However, doses of 10 and 30 mg/kg reached their maximum effect at 60 min, and in particular, these doses exhibited fluctuations in the magnitude of the response throughout the experimental period, where at 10 and 30 mg/kg variations were observed between 120 and 240 min; at 300 mg/kg, the maximum effect observed at 60 min was maintained throughout the evaluation period. This effect was comparable to that observed with GBP, which produced a significant effect starting at 60 min and reached its maximum magnitude at 90 min, remaining in effect for the 240 min of evaluation in a similar manner to that observed in female rats (Treatment: F_5,270_ = 86.61, *p* < 0.0001; Time: F_8,270_ = 16.01, *p* < 0.0001; Interaction: F_40,270_ = 2.892, *p* < 0.0001) (Figure 4B).

The AUC_0–240 min_ from the temporal course curves showed that the cumulative time response of flinching/licking time increased significantly and similarly in female and male rats treated with reserpine (Figure 4C). These responses were equivalent and produced a significant antiallodynic effect at all doses of the extract tested in females and males as 10 mg/kg (63.01 ± 4.74% and 58.87 ± 5.85%), 30 mg/kg (64.05 ± 11.35% and 68.00 ± 14.51%), and 300 mg/kg (53.83 ± 7.16% and 67.17 ± 7.48%), and similarly to that presented with GBP (59.94 ± 7.99% and 68.13%), respectively (Treatment: F_5,60_ = 47.57, *p* < 0.0001; Sex: F_1,60_ = 0.8742, *p* = 0.3535; Interaction: F_5,60_ = 0.4682, *p* = 0.7984) (Figure 4C).

### 2.4. Antihyperalgesic Effect of the Hexane Extract of the Aerial Parts of S. rosmarinus on Reserpine-Induced Mechanical Hyperalgesia in Female and Male Rats

Regarding the antihyperalgesic effect, a dose response was observed in female rats since the lowest dose of 10 mg/kg of *S. rosmarinus* showed a significant response from the first 60 min and remained at this level until the end of the evaluation in comparison to the effect observed in the reserpine group. This antihyperalgesic effect was improved when the doses were increased to 30 mg/kg and 300 mg/kg. The effect was evident starting 30 min after administration reached its maximum intensity and was maintained throughout the 240 min evaluation period (Figure 5A). The effect of GBP (30 mg/kg) was similar to the lowest dose of the extract administration, which produced a significant antihyperalgesic effect from 30 min onwards but showed a delay in reaching the maximum effect observed in the presence of the extract (Treatment: F_5,270_ = 86.12, *p* < 0.0001; Time: F_8,270_ = 9.79, *p* < 0.0001; Interaction: F_40,270_ = 2.49, *p* < 0.0001) (Figure 5A).

In the case of male rats, the extract at 10 and 30 mg/kg did not produce a significant antihyperalgesic effect; until the dose was increased to 300 mg/kg, a significant response was evident 30 min after administration, reaching its maximum magnitude at that same moment and remaining throughout the evaluation period. This effect was numerically greater than that observed with GBP (30 mg/kg), which had a significant antihyperalgesic effect that started at 150 min and was maintained throughout the rest of the experiment (Treatment: F_5,270_ = 89.64, *p* < 0.0001; Time: F_8,270_ = 5.91, *p* < 0.0001; Interaction: F_40,270_ = 1.59, *p* = 0.0182) (Figure 5B).

The AUC_0–240 min_ from the time course curves showed that muscle pressure was significantly and similarly reduced in female and male rats treated with reserpine (Figure 5C). The 10 mg/kg extract did not produce a significant antihyperalgesic effect in any of the rats (37.68 *±* 3.22% and 6.12 ± 0.32%). In contrast, a significant response was observed in female rats when the extract dose was increased to 30 mg/kg (72.19 *±* 2.33%) and 300 mg/kg (82.13 *±* 2.92%). Male rats also achieved a significant effect, but only with the 300 mg/kg of the extract (82.13 *±* 4.65%) (Treatment: F_5,60_ = 33.38, *p* < 0.0001; Sex: F_8,270_ = 4.774, *p* = 0.0328; Interaction: F_5,60_ = 2.098, *p* = 0.078) (Figure 5C). The significant response to the extract in females (30 mg/kg) or males (300 mg/kg) was greater than that produced by the reference drug GBP (30 mg/kg, 37.68 *±* 1.57%) (t_9.644_, = 2.540, *p* = 0. 0301 or t_9.975_, = 2.384, *p* = 0. 0384, respectively) (Figure 5C).

### 2.5. Participation of the Estrogen Receptor β in the Antihyperalgesic and Antiallodynic Effects of a Non-Polar Extract of S. rosmarinus in the FM-like Pain Model in Female Rats

The time course curve shows that administration of PHTPP (25 µg/rat) did not produce an anti-allodynic effect per se on tactile allodynia throughout the experimental analysis. However, the significant effect produced by the Hex-Ext (30 mg/kg, i.p.) from the first 30 min and maintained during the 240 min of the evaluation period was totally prevented by PHTPP (25 µg/rat), as no anti-allodynic effect was observed (Treatment: F_4,225_ = 273.10, *p* < 0.0001; Time: F_8,225_ = 4.10, *p* = 0.0001; Interaction: F_32,225_ = 2.82, *p* < 0.0001) (Figure 6A). The time course of AUC_0–240 min_ shows that the administration of PHTPP (25 µg/rat) produced no effect, but its presence inhibited the significant antiallodynic effect observed preliminarily with the extract alone at 30 mg/kg (74.93 ± 3.58%), which was reduced to 13.81 ± 2.58% (F_4.25_ = 71.88, *p* < 0.0001) (Figure 6B).

In thermal allodynia, the administration of PHTPP (25 µg/rat) did not produce an antiallodynic effect per se. However, the significant antiallodynic response induced by the *S. rosmarinus* extract at a dose of 30 mg/kg in thermal allodynia, starting 60 min after administration and lasting for the 240 min of evaluation, was partially reduced (Treatment: F_4,225_ = 156.90, *p* < 0.0001; Time: F_8,225_ = 4.85, *p* < 0.0001; Interaction: F_32,225_ = 2.81, *p* < 0.0001) (Figure 6C). The temporal evolution of the AUC_0–240 min_ reinforces that the administration of PHTPP (25 µg/rat) did not produce an antiallodynic effect. However, it reduced the significant response of the 30 mg/kg of the extract from 64.05 ± 11.35% to 49.45 ± 6.95% (F_4,25_ = 28.40, *p* < 0.0001) (Figure 6D).

As it was observed in tactile and thermal allodynia, the administration of PHTPP (25 µg/rat) did not produce an antihyperalgesic effect on its own. However, in a similar manner to tactile allodynia, it completely inhibited the significant antihyperalgesic response of *S. rosmarinus* extract (30 mg/kg, i.p.) in female rats. (Treatment: F_4,225_ = 251.60, *p* < 0.0001; Time: F_8,225_ = 3.69, *p* = 0.0005; Interaction: F_32,225_ = 3.17, *p* < 0.0001) (Figure 6E). The AUC_0–240 min_ of the time course shows that the administration of PHTPP (25 µg/rat) did not produce an antihyperalgesic effect, but the antihyperalgesic effect produced by the 30 mg/kg of the *S. rosmarinus* extract (72.19 ± 2.33%) was completely inhibited by this antagonist (F_4.25_ = 67.93, *p* < 0.0001) (Figure 6F).

### 2.6. Participation of the Androgen Receptor in the Antihyperalgesic and Antiallodynic Effects of a Non-Polar Extract of S. rosmarinus in the FM-like Pain Model in Male Rats

In the time course of tactile allodynia evaluated in males, it was observed that the administration of flutamide (10 mg/kg) produced no effect on its own during the entire evaluation. However, its presence caused the significant effect of the *S. rosmarinus* extract to have a shorter latency, and the anti-allodynic response was significant from 1 h prior, further enhancing the effect, which reached its maximum at 180 min and persisted until the end of the evaluation (Treatment: F_4,225_ = 269.60, *p* < 0.0001; Time: F_8,225_ = 3.67, *p* = 0.0005; Interaction: F_32,225_ = 4.87, *p* < 0.0001) (Figure 7A). The AUC_0–240 min_ from these time courses allows us to observe no response after flutamide administration (10 mg/kg, i.p.) on its own. Nevertheless, the significant antiallodynic response of 30 mg/kg (18.34 ± 2.04%) was almost duplicated in male rats that received pretreatment with flutamide (10 mg/kg) until 31.28 ± 2.39% (F_4.25_ = 118.40, *p* < 0.0001) (Figure 7B).

In the case of thermal allodynia, the administration of flutamide (10 mg/kg) also did not produce an anti-allodynic effect per se. Interestingly, when male rats received the S. rosmarinus extract at 30 mg/kg, i.p. and pretreatment with flutamide (10 mg/kg) resulted in a significant anti-allodyinic effect that was maintained until the end of the evaluation, rather than a progressive decrease in this response in the last hour of measurement in male rats receiving the extract only (Treatment: F_4,225_ = 198.10, *p* < 0.0001; time: F_8,225_ = 5.00, *p* < 0.0001; interaction: F_32,225_ = 3.44, *p* < 0.0001) (Figure 7C). The AUC_0–240 min_ obtained from the time curves shows that administration of flutamide (10 mg/kg) alone did not cause any effect alone, and although changes in latency and time to maximum effect are observed over the time course curves, the expression of the AUC_0–240 min_ indicates that the antiallodynic effect of the *S. rosmarinus* extract (30 mg/kg, i.p.) of 68.00 ± 14.51% was maintained at 68.42 ± 11.46% in the presence of the antagonist (F_4,25_ = 32.11, *p* < 0.0001) (Figure 7D).

Similar to that observed in tactile allodynia, administration of flutamide (10 mg/kg) did not produce changes in the mechanical hyperalgesia response, but its pretreatment in male rats administered with *S. rosmarinus* extract (30 mg/kg) enhanced the significant antihyperalgesic effect (Figure 7E) (Treatment: F_4,225_ = 148.20, *p* < 0.0001; Time: F_8,225_ = 4.16, *p* = 0.0001; Interaction: F_32,225_ = 1.61, *p* = 0.0256). The AUC_0–240 min_ confirms that flutamide (10 mg/kg) does not produce an effect by itself but improves the significance of the preliminary antihyperalgesic effect (54.05 ± 0.92%) produced by the extract at a dose of 30 mg/kg (31.44 ± 3.27%) (F_4,25_ = 27.44, *p* < 0.0001) (Figure 7F).

## 3. Discussion

Non-polar extracts such as the essential oil of *S. rosmarinus* are included in herbal formulations for human use, suggesting their potential properties against nociplastic or FM-like pain. The hexane extract resembles bioactive essential oils prepared with *S. rosmarinus* batches from all over the world, as monoterpenes (namely 1,8-cineol, camphor, and α- and β-pinene) are considered chemical markers in such bioactive extracts [22]. In this study, the antiallodynic and antihyperalgesic effects of the aerial parts of *S. rosmarinus* prepared as a non-polar hexane extract were evaluated in the FM-like pain because of the differential efficacy detected by separating female and male rats; additionally, the participation of androgen and estrogen receptors was determined as part of the mechanisms of action of this medicinal plant used to relieve pain, reinforcing its wide spectrum, not only for the well-known functional antinociceptive activity but also for dysfunctional and nociplastic pain diseases.

FM is a chronic pain syndrome characterized by widespread musculoskeletal pain associated with extreme fatigue, sleep disturbances, and cognitive issues [1]. Scientists believe the brain amplifies pain signals; while there is no cure, the focus is on finding comprehensive treatments to improve quality of life, including natural products with antioxidant properties such as *S. rosmarinus* [21,23,24]. The widespread pain associated with FM highlights two key diagnostic symptoms: allodynia, where harmless stimuli (such as the touch of clothing) cause pain, and hyperalgesia, an exaggerated and severe response to a stimulus that would normally cause only mild discomfort [1,25]. It is also important to note that FM predominantly affects women (accounting for up to 80% of cases [4], though men also suffer from it; while the condition is similar, it may manifest with certain differences [26]. Thus, the aim of this project was to evaluate the efficacy of a non-polar *S. rosmarinus* extract—a plant noted in traditional medicine for pain relief [27]—in alleviating pain-related behaviors such as allodynia and hyperalgesia in male and female rats. This was achieved by inducing FM-like pain through the subcutaneous administration of reserpine (1 mg/kg) over three days, which lowered the mechanical stimulation threshold—assessed via von Frey filaments and thermic tactile allodynia. Additionally, mechanical hyperalgesia was measured using the Randall-Selitto apparatus, while cold sensitivity was determined via the acetone test, a condition characterized as thermal allodynia. Collectively, these behaviors confirm the successful establishment of the FM-like pain model [28,29]. Although the literature reports that repeated reserpine administration induces both characteristic nociceptive behaviors of FM-like pain in a similar degree in male and female rats—reproducing a state of generalized, persistent pain [30]—it remains unknown whether treatments might generate sex-related differences in pain relief.

The reason why reserpine administration induces generalized pain is that this treatment regimen causes the depletion of biogenic amines—primarily dopamine, noradrenaline, and serotonin (5-hydroxytryptamine, 5-HT)—through the inhibition of the vesicular monoamine transporter (VMAT) in CNS structures such as the spinal cord, thalamus, and prefrontal cortex [31]. Given that these neurotransmitters play a fundamental role in descending pain-inhibitory pathways, their reduction promotes altered nociceptive processing and the development of central sensitization phenomena, which are considered key mechanisms in the pathophysiology of FM [32]. In this regard, the reduction in thresholds observed in the present study supports the validity of the model used and confirms that the animals developed a state of hypersensitivity consistent with the sensory alterations described in FM patients, thereby establishing the model as a suitable tool for evaluating the potential of *S. rosmarinus* for this kind of pain.

Once the FM-like pain model was established, intraperitoneal administration of the non-polar *S. rosmarinus* extract significantly reduced reserpine-induced tactile allodynia, thermal allodynia, and mechanical hyperalgesia, demonstrating these effects in both sexes. Overall, these results indicate that the non-polar extract can attenuate the state of hypersensitivity and allodynia characteristic of this model and of FM-like pain [28,29,30,32]. However, it should be noted that the magnitude of the effects varied significantly depending on the behavioral test, the administered dose, and the sex of the animals. The observed efficacy was comparable to, or even greater than, that of the reference drug GBP, depending on the experimental conditions. These findings highlight the pharmacological potential of this non-polar *S. rosmarinus* extract as a source of compounds with analgesic activity against nociplastic pain. These results align with studies attributing antinociceptive, anti-inflammatory, and antioxidant properties to this plant—properties associated with monoterpenes, diterpenes, and triterpenes found in the plant’s lipophilic fraction [23].

Sex differences observed in the pharmacological effects of *S. rosmarinus* were related to the antiallodynic and antihyperalgesic behavioral responses evaluated in the reserpine-induced FM-like pain. All doses of *S. rosmarinus* were effective in males and females in thermal-allodynic response in the same range of dose and magnitude of effect, in contrast to the response observed in tactile allodynia and mechanical hyperalgesia, where female rats were more sensitive to the effects of *S. rosmarinus* than males. In this FM-like pain model, Transient Receptor Potential A1 (TRPA1) activation has been shown to contribute to the onset of both mechanical and thermal allodynia, and its pharmacological blockade significantly reduces both nociceptive responses [33]. Interestingly, TRPA1 could be activated by an ERβ agonist [34]; however, PHTPP administration did not cancel the antiallodynic effect of the extract, neither in females nor in males, suggesting participation of another mechanism of action. Several reports indicate a crosstalk between estrogen receptors; in fact, ERβ could be repressed by the activation of ERα [14,35]. Further, it was reported that the antiallodynic effect of AC 186, a pure agonist to ERβ, is canceled by the antagonism of ERα [36]. Therefore, it is possible that some of the compounds reported in the extract of *S. rosmarinus* that possess affinity for ERα could induce their anti-allodynic effect via TRPA1 through ERα receptor modulation. According to the GC-MS analysis of the *S. officinalis* hexane extract, at least 16 constituents were detected, such as (+)-2-Bornanone (camphor) as the most abundant; this monoterpene has been reported to activate and desensitize the TRPV1 channel through a vanilloid site-independent mechanism, as well as inhibit TRPA1 in a murine model of neuropathic pain [37], where camphor reduced hyperalgesia and hyperexcitability of dorsal root ganglion neurons, an effect attributed mainly to blocking TRPA1-mediated responses [38]. In a similar manner, borneol has been shown to inhibit TRPA1-mediated cationic currents in trigeminal ganglion neurons and to reduce responses induced by nicotine, an agonist of this channel [39], thereby reducing mechanical hyperalgesia in murine models of chronic neuropathic and inflammatory pain [40]. Whereas in the case of 1,8-cineole, an inhibition of TRPA1 channels was related to nociception and thermal sensitivity [41]. Finally, camphene reduced thermal and mechanical hyperalgesia by inhibiting CaV3.2 T-type calcium channels, an effect observed in both females and males with no difference [42]. Meanwhile, β-caryophyllene, considered an agonist of the CB_2_ cannabinoid receptor, reduced nociceptive behaviors in rats, with the effect being more pronounced in males [43]. Then, it is possible that TRPA1 participates in the anti-allodynic and antihyperalgesic effects induced by this non-polar extract of *S. rosmarinus* in females and males.

It has been reported that anti-hyperalgesic and antiallodynic (thermal and tactile) responses in males could be mediated by androgen receptors. Following this idea, flutamide, an unspecific AR antagonist, was evaluated in the presence of the extract in male rats. It was observed that flutamide did not block the effect of the extract; on the contrary, the antagonist increased the analgesic response of *S. rosmarinus*, suggesting that androgen induction of the analgesic response might be mediated by the conversion of androgen to estrogen [44]. A limitation is that participation of estrogen receptors was not explored in the effect of the *S. rosmarinus* extract in males. However, the literature reports that estrogens, as androgenic metabolites, mediate neuroprotective effects on N27 (for female) and PC12 (for male) neuronal cells [45] and the antidepressant-like action of compounds that act on the serotonergic system and noradrenergic system [46], both involved in FM-like pain modulation (reviewed in [47]). Future specific experiments using an aromatase inhibitor such as formestane (4-hydroxyandrostenedione) may help corroborate this proposal.

Regarding female rats, the antagonist to ERβ inhibited the effect of *S. rosmarinus* in tactile allodynia and mechanical hyperalgesia, suggesting the participation of ERβ in the effect of its non-polar extract. For ERα, some active compounds present in this extract are consistently reported to have affinity for it and for androgen receptors [20,21]; however, for ERβ, evidence is sparse; for example, two estrogen receptor-β agonists, ERB-041 and AC-186, blocked hyperalgesia and allodynia in models of inflammatory and chemical-induced pain [48]. Then, it is possible that both receptors, ERα and ERβ, participate in the extract’s effects. One possibility to explain the apparent discrepancy derived from the effect of PHTPP that canceled the action of *S. rosmarinus* in two models arises from the fact that some of these compounds could be acting as phytoestrogens on ERα, which in turn may contribute to the action of ERβ via crosstalk [12,14] and promote the cancelation of its effect, as has been proposed for AMC 186, a pure agonist to ERβ, that could be inactivated by both ERα and ERβ antagonism [36]. If this is true, the antagonism of ERα also could cancel the effect of the extract of *S. rosmarinus* in both sexes. Future experiments could confirm this proposal.

Another non-exclusive explanation of why the effect of *S. rosmarinus* could be canceled by ERβ lies in the relation of estrogen and the serotonergic system. An involvement of estrogens in the regulation of the reserpine-induced FM-like pain model in rats was preliminarily reported [29,49], where an estrogen restitution in ovariectomized rats produced robust anti-hyperalgesic and anti-allodynic effects [29]; therefore, it is likely that some compounds in *S. rosmarinus* act as phytoestrogens [50], as their effects are canceled by the antagonism of ERβ. Estrogens and phytoestrogens modulate the activity of the serotonergic system at several levels [47], mainly via ERβ activation, for example, in the 5-HT_1A_ [50] and 5-HT_2A_ receptors [49] and tryptophan hydroxylase regulation [51], involved in FM regulation [47,49]. Another mechanism likely associated with the effect of *S. rosmarinus* in the FM-like pain model mediated by ERβ might involve the P2X3 receptor, which has a relevant role in nociception and inflammation upon activation by adenosine triphosphate (ATP). The ERβ agonist promotes downregulation of the P2X3 receptor, which in turn decreases c-Fos and ERK, exerting anti-hyperalgesic effects [52]. Therefore, the extract of *S. rosmarinus* via ERβ could be acting on P2X3 and TRPA1 to induce anti-hyperalgesic and anti-allodynic actions in FM-like pain in male and female reserpine-treated rats. However, further studies are needed in the future to strengthen the involvement of these possible mechanisms in the effect of *S. rosmarinus* for the treatment of FM-type pain.

These results reinforce previous research demonstrating the antinociceptive activity of *S. rosmarinus* in various experimental pain models—acting at local, peripheral, and central levels—and expand knowledge regarding its spectrum of activity. Extracts from this plant species have significantly reduced nociceptive behaviors in different models of chemical and inflammatory pain [16], where major triterpenes found in this plant—specifically ursolic acid and oleanolic acid—contributed significantly to its antinociceptive effect in those types of pain [52]. Although those studies utilized different pain models from the one employed in the present work, the results support the hypothesis that lipophilic metabolites present in the *S. rosmarinus* non-polar extract may play a role in reducing mechanical sensitivity in FM-like pain. Both with affinity to estrogens and androgen receptors. Tactile allodynia is a key manifestation of the central sensitization characteristic of FM, as it reflects a painful response to mechanical stimuli that does not elicit pain under physiological conditions. Administration of this extract significantly reduced reserpine-induced tactile allodynia in rats of both sexes; this indicates that the extract’s bioactive components can modulate pain-processing mechanisms involving neurochemical alterations in serotonin, noradrenaline, and dopamine neurotransmitter systems—neurotransmitters essential for the function of descending pain-inhibitory pathways [32,53]. Consequently, the recovery of the mechanical threshold observed following extract administration suggests an attenuation of the reserpine-induced state of central sensitization. However, this suggests a different mechanism of action since females respond to lower doses than males.

A particularly significant finding of this project was the marked difference in the pharmacological response profile between males and females. While the 30 mg/kg dose elicited the greatest antiallodynic effect in female rats, maximum efficacy in males was observed only at a dose of 300 mg/kg; this demonstrates that females are more sensitive to the non-polar constituents in *S. rosmarinus* extract than males. Depending on the type of stimulus (tactile vs. thermal vs. mechanical), this finding is of particular interest because, as previously noted, FM is more prevalent in women, and the mechanisms governing pain processing and modulation differ between the sexes. Sex hormones—particularly estrogens—modulate the activity of serotonergic, noradrenergic, and dopaminergic systems and influence inflammatory responses and neuronal excitability; these factors are closely linked to the development and maintenance of central sensitization [54]. Recent studies indicate that sex-specific neuroimmune mechanisms play a role in the persistence of chronic pain: spinal microglia activation predominates in males, whereas T lymphocytes and other adaptive immune cells play a greater role in females [7]. Such differences could alter responses to compounds with anti-inflammatory, antioxidant, and neuromodulatory properties—such as those found in *S. rosmarinus*—thereby helping to explain the divergent efficacy profiles observed between females and males in the present study. However, since the mechanisms underlying these differences were not directly assessed, this interpretation should be viewed as a hypothesis requiring confirmation through further research aimed at elucidating the pharmacological and molecular bases of the extract’s antiallodynic and antihyperalgesic effects.

The pharmacological activity of *S. rosmarinus* extracts in the FM-like pain model is therefore associated in part with its non-polar chemical composition, as it primarily contains lipophilic compounds such as carnosic acid, carnosol, ursolic acid, oleanolic acid, and various monoterpenes and sesquiterpenes. These metabolites have been shown to possess antioxidant, anti-inflammatory, neuroprotective, and antinociceptive properties by decreasing pro-inflammatory cytokines, reducing oxidative stress, and modulating signaling systems involved in nociceptive transmission [23,55]—mechanisms that could collectively contribute to reducing pain-like behaviors induced in FM-like pain in male or female rats, while also acknowledging that a hormonal component might be relevant and of interest for elucidating the mechanism of action underlying its pharmacological activity. Administration of the 30 mg/kg dose of the extract resulted in an onset of effect in female rats characterized by a shorter latency and greater intensity than that observed with gabapentin during the mechanical allodynia assessment period; in contrast, in males, the 300 mg/kg dose scarcely achieved efficacy comparable to the reference drug. Although these results do not establish the extract’s therapeutic superiority over GBP—given that the latter was administered at 30 mg/kg (i.p.)—they do highlight the pharmacological potential of *S. rosmarinus* as a source of bioactive compounds potentially useful for treating FM-associated pain. These findings expand upon the scientific evidence previously generated by the research group by demonstrating that the non-polar extract of *S. rosmarinus*—a plant used in traditional medicine—exerts anti-allodynic activity in a model of FM-like pain. Furthermore, this response is sex-dependent, favoring females—a demographic with higher FM prevalence and incidence rates that had not been explored in previous studies. In contrast to tactile allodynia, the non-polar extract of *S. rosmarinus* produced a consistent anti-allodynic effect against thermal stimuli in both sexes. Data from both the time-course analysis and the area under the curve showed an equivalent effect; all three tested doses significantly reduced reserpine-induced thermal allodynia, demonstrating efficacy comparable among themselves and similar to that observed with GBP. No dose–response relationship was observed regarding the effect, as a plateau was reached at the lowest dose and did not change significantly with increasing doses. This behavior suggests that the mechanisms involved in thermal allodynia are highly sensitive to the metabolites present in the extract.

Finally, this non-polar extract of *S. rosmarinus* reduced mechanical hyperalgesia in a dose-dependent manner, particularly in female rats. Analysis of the time course and the area under the curve revealed that doses of 30 and 300 mg/kg produced a significant effect; the 300 mg/kg dose demonstrated the greatest efficacy, showing a percentage of inhibition higher than that observed with the reference drug, GBP. In contrast, among male rats, only the 300 mg/kg dose produced a significant reduction, as the 30 mg/kg dose showed merely a trend toward decreasing the hyperalgesia, and the 10 mg/kg dose showed no difference compared to the group treated with reserpine and vehicle. These results indicate the existence of sex-related differences in the antihyperalgesic response regarding treatment sensitivity. The mechanical hyperalgesia observed following reserpine administration stems from a central sensitization process driven by the depletion of monoamines involved in the descending modulation of pain. Reserpine treatment has been shown to reduce monoamine levels in CNS regions associated with nociceptive processing, thereby inducing a persistent state of neuronal hyperexcitability characterized by a lowered mechanical threshold and an enhanced response to noxious stimuli [32]. This phenomenon aligns with the mechanisms described in FM, which involves central amplification of pain signals associated with altered glutamatergic transmission, N-Methyl-D-Aspartate (NMDA) receptor activation, and plastic changes in nociceptive pathways [56]. In addition to neuronal changes, neuroinflammation plays a significant role in maintaining mechanical hypersensitivity. The activation of glial cells—particularly microglia and astrocytes—promotes the release of proinflammatory mediators such as IL-1β and TNF-α, as well as neurotrophic factors; these substances increase the excitability of nociceptive neurons and sustain the state of central sensitization [57]. In this context, the antihyperalgesic activity of this non-polar extract of *S. rosmarinus* may be linked to the ability of its bioactive metabolites to modulate several processes associated with inflammation and oxidative stress.

The evidence found in this study suggests the involvement of hormonal factors in the extract’s antihyperalgesic response. Female rats showed greater sensitivity to the treatment, as intermediate doses (30 mg/kg) were sufficient to produce a significant effect, whereas males required the highest dose (300 mg/kg) to achieve a comparable response. These differences align with evidence demonstrating that the mechanisms involved in pain perception and modulation vary between the sexes. Factors such as sex hormones, immune cell activity, glial signaling, and differences in neurotransmission have been shown to modify both pain sensitivity and the response to analgesic treatments [7,54]. Specifically, it has been proposed that spinal microglia play a predominant role in pain sensitization in males, whereas mechanisms mediated by adaptive immune cells—such as T lymphocytes—are more significant in females [7]. These differences could influence the response to compounds found in *S. rosmarinus* that possess anti-inflammatory and antioxidant properties, resulting in distinct pharmacological profiles between the sexes. However, further studies are needed to evaluate and characterize the metabolites responsible for this effect and to determine the molecular targets involved in modulating peripheral and central sensitization.

The evidence of the antiallodynic and antihyperalgesic effects of *S. rosmarinus* differing between female and male rats is relevant for understanding sex-related differences in FM-type pain. However, the reproducibility of the effects observed in both sexes of Wistar rats in the reserpine-induced FM-like pain model needs to be strengthened in other strains, species, or experimental models of nociplastic pain, as well as in clinical studies to determine its translational significance.

## 4. Materials and Methods

### 4.1. Plant Material

The aerial parts of *S. rosmarinus* Spenn. (formerly known as *R. officinalis* L.) were acquired at the Sonora Market, Mexico City, on October 18, 2024. The specimen was authenticated by taxonomist Dr. Martha Gordillo and conserved as a reference in the herbarium of the Facultad de Ciencias, Universidad Nacional Autónoma de México, under receipt number 185759.

### 4.2. Extract Preparation

The dried aerial parts of *S. rosmarinus* (885 kg) were cut into small pieces and placed in 3 L of n-hexane using a container for maceration during 72 h × 3 times. After filtration and complete evaporation of solvent using a rotatory evaporator (RE301; Yamato rotatory evaporator, Tokyo, Japan), a final product yield of 1.86% (16.43 g) was obtained.

### 4.3. GC-MS Chromatographic Analysis

The hexane extract of *S. rosmarinus* was analyzed using an Agilent 7890B gas chromatography system coupled to an Agilent 5977A mass spectrometer (Agilent Technologies, Santa Clara, California (CA), United States of America (USA)). Compound separation was performed using an HP-5MS column (30 m × 0.25 mm × 0.25 μm). The oven program was configured as follows: initial temperature at 40 °C, held for 1 min, increased at 8 °C/min up to 310 °C, held for 8.25 min, and brought to equilibrium for 0.5 min at 350 °C. The extract was dissolved in GC-MS grade hexane and injected in split mode (20:1) at 280 °C. Helium at a constant flow rate of 1 mL/min was used as carrier gas. The electroionization potential was set to 70 eV, and a quadrupole mass analyzer was configured in SCAN mode, acquiring spectra from *m*/*z* 50 to 500. Data processing was performed using MassHunter Workstation Software (Unknown Analysis and Qualitative Analysis modules, v. B.07.00; Agilent Technologies, Santa Clara, CA, USA). Compound identification was performed by comparison with the NIST 14 mass spectrum library.

### 4.4. Animals

Wistar rats (200–230 g body weight, 7–8 weeks old, both sexes) provided by the vivarium of the Instituto Nacional de Psiquiatría Ramón de la Fuente Muñiz (INPRFM) were used in groups of at least 6 animals. The animals were kept in acrylic cages with ad libitum access to food and water in a temperature-controlled room (22 ± 2 °C) and under a 12 h light/dark cycle (light on at 7:00 a.m. and off at 7:00 p.m.) until the start of the experiments. No additional environmental enrichment was provided. All procedures were performed by trained personnel, and the animals were habituated to the experimental conditions for three days before testing to minimize stress. The animals were monitored daily throughout the experimental period to assess their overall health, behavioral changes, hypoactivity, body weight loss, and access to food and water. Hypoactivity and body weight loss were expected effects associated with reserpine administration; however, these remained within predefined acceptable limits, and no unexpected adverse events were observed. Humane endpoint criteria were established a priori, including signs of severe or persistent distress, body weight loss exceeding 20%, or inability to access food or water.

The studies were carried out in accordance with the guidelines of the local institution’s bioethics committee, as well as national Norma Oficial Mexicana (NOM-062-ZOO-1999) and international regulations for the care and use of laboratory animals. This research was approved by the Research Committee of the INPRFM (CEI/C/035/2018, 4 June 2018) and reapproved by CEI/C/035/2018/2026 (12 January 2026), protocols NC123280.0 (10 September 2012) and NC17073.0 (12 December 2017).

### 4.5. Drugs and Reagents

The 4-[2-phenyl-5,7-bis(trifluoromethyl)pyrazolo [1,5-α]pyrimidine-3-yl]phenol (PHTPP) (β-estrogen receptor antagonist), flutamide (non-steroidal antiandrogenic drug), gabapentin (GBP, reference drug), reserpine, acetic acid, and Tween-80 were purchased from Sigma-Aldrich (St. Louis, Missouri (MO), USA).

Reserpine was dissolved in 0.5% acetic acid and administered subcutaneously (s.c.) at a dosage of 1 mg/kg. All treatments, including reference drug GBP (30 mg/kg, i.p.) [58], were prepared fresh on the day of the experiment by either dissolving or resuspending the extract in 0.2% tween 80 to intraperitoneally (i.p.) administrate 10, 30, and 300 mg/kg, respectively. Treatments were administered in a volume of 0.1 mL/100 g of body weight. Flutamide was administered using almond oil 10 mg/kg, s.c. [59]. PHTPP was dissolved in 5% dimethyl sulfoxide (DMSO) in saline solution and subcutaneously administered at 25 μg/rat [60].

### 4.6. Experimental Design

Experimental FM-type pain was induced according to a modified [30] previous method described [32]. Ninety-six rats (forty-eight females and forty-eight males) were habituated for three days prior to induction and to the measurements of hyperalgesia and allodynia. Reserpine was administered subcutaneously in the back of the neck once daily for three consecutive days, at a volume of 1 mL/kg (see timeline Figure 8).

The design included 16 experimental groups of at least n = 6 rats each randomly assigned (see time-line Figure 8). The acute treatments (single dose) were the following in each female or male subject:

**Stage 1**

- Group 1 or 2: Naïve female or male rats.
- Group 3 or 4: Reserpinized female or male rats (reserpine, 1 mg/kg, s.c., 3 times).
- Group 5 or 6: Reserpine + GBP (30 mg/kg, i.p.) female or male rats.
- Groups 7, 9, and 11 or 8, 10, and 12: Reserpine + Hex-Ext (10, 30, and 300 mg/kg, i.p.) female or male rats, respectively.

**Stage 2**

And additional groups were also explored to determine a possible mechanism of action involved, such as estrogens and androgens antagonism alone and on the antiallodynic and antihyperalgesic effect shown by the animals with the administration of Hex-Ext:

- Group 13: PHTPP (25 µg/rat 10 mg/kg) in female rats.
- Group 14: Flutamide (10 mg/kg) in male rats.
- Group 15: PHTPP (25 µg/rat) + Hex-Ext (30 mg/kg) in female rats.
- Group 16: Flutamide (10 mg/kg) + Hex-Ext (30 mg/kg) in male rats.

Flutamide was administered for 14 days prior to the test [57]. PHTPP was administered 24 h before starting the test [61]. Treatments were administered on the 5th day after induction, when maximum nociception was detected based on hyperalgesia and allodynia thresholds [30,32]. All treatments were evaluated after 30 min of administration and over a 4 h time course, with measurements at 0, 30, 60, 120-, 150-, 180-, and 240 min post-administration. Data from the time curves were converted to AUC to determine efficacy.

Inclusion and exclusion criteria were established a priori based on the development of allodynic and hyperalgesic responses following reserpine administration. Animals exhibiting predetermined thresholds for allodynia and hyperalgesia on the fifth day post-induction were included. Animals that did not reach these thresholds were excluded from the experimental groups. The model showed an approximate success rate of 80% in female rats and 70% in male rats, based on the development of predetermined thresholds for allodynia and hyperalgesia. No animals or data meeting the inclusion criteria were subsequently excluded from the statistical analyses.

To minimize potential confounding factors, all animals were kept in the same housing conditions and habituated to the experimental conditions for three days prior to testing. All behavioral assessments were conducted in the same laboratory, during the same period (8:00 a.m. to 2:00 p.m.), and by the same researchers throughout the study. Female and male rats were assessed separately to avoid potential interference associated with the simultaneous presence of animals of both sexes. In all tests and in all behavioral trials, the stimulus was always applied to the right hind limb. For the blinding of the study, the researcher who carried out the experiments was aware of the random assignment of the groups, while in the experimental procedure it was carried out by two researchers (one in charge of administering the groups and another of measuring the thresholds); the data are recorded using symbols in such a way that the researchers who carry out the test do not know the values of the behavioral responses until recording the results and subsequently analyzing the data.

### 4.7. Assessment of Tactile Allodynia

The tactile response threshold was calibrated using Von Frey filaments (Stoelting Co., Ltd., Wood Dale, Illinois (IL), USA) with the up-and-down method. Rats were placed individually in transparent acrylic boxes without a bottom lid, on a wire mesh on an elevated platform, exposing the sole of the limb. Subsequently, the mid-portion of the right hind limb was stimulated with calibrated von Frey filaments (2, 4, 6, 8, 10, and 15 g).

A positive response was defined as limb withdrawal within a period of up to 6 s; a negative response was defined as no response at that time. Negative responses were marked with an “O” and positive responses with an “X”. After obtaining the first positive response, testing continued with the immediately smaller filament; if the new response was negative, the smaller filament was tested; if it was positive, the next larger filament was used, until a pattern of five measurements was completed, ranging from positive (X) to negative (O).

The paw withdrawal threshold was calculated as the tactile response threshold using an adaptation of Dixon’s top-down paradigm [62]. The 50% withdrawal threshold was calculated using the formula as follows [63]:

(1) $$50\%\;\mathrm{umbral}(g)=\frac{10^{\left[X_{f}+\kappa \delta \right]}}{10,000}$$

where

- **X_f_**: value of the last von Frey filament used (in logarithmic units).
- **κ**: correction factor based on the response pattern (according to the calibration table and the number of positive and negative responses).
- **δ**: average difference between stimuli (in logarithmic units).

### 4.8. Thermal Stimulus Threshold (Thermal Allodynia)

Thermal allodynia to a cold stimulus was assessed using the acetone test. For this test, each rat was individually placed into a transparent acrylic cylinder, which was located on a wire rig. A volume of 50 μL of acetone was then applied to the mid-dorsal surface of the right hind paw using a drip adapter on an insulin syringe. Immediately afterward, the licking or/and shaking time of their right hind paw was recorded for 60 s in each rat. This test was performed in triplicate, with a 5 min interval between each measurement, and the average value obtained was considered as the thermal allodynia threshold.

### 4.9. Mechanical Hyperalgesia Assessment

Muscle pressure threshold was assessed using the Randall–Selitto apparatus (Ugo-Basile, Varese, Italy). For this assessment, rats were immobilized with a cloth, leaving the right hind paw exposed, and increasing linear pressure was applied to the medial gastrocnemius muscle (maximum force: 250 g). A positive response was considered when the rats withdrew their limb or vocalized. Each study was performed in triplicate, with a minimum inter-stimulus interval of 1 min [32,61].

### 4.10. Statistical Analysis

Data are expressed as the mean ± SEM of n = 6 repetitions. Data normality was assessed using the Shapiro–Wilk test, and all datasets met the normality assumption. Time-course curves were analyzed using repeated measures in a two-way analysis of variance (ANOVA) followed by Tukey’s post hoc test. The area under the curve (AUC) was calculated using the trapezoidal method, and data were analyzed using a one-way ANOVA followed by Tukey’s post hoc test to compare the mean of each group with the mean of all other groups, or Student’s *t*-test between specific groups. Statistical analysis was performed using GraphPad Prism version 10.5.0 for Windows (GraphPad Software INC, La Jolla, CA, USA). A *p* < 0.05 was considered to declare a significant difference.

## 5. Conclusions

A non-polar extract of *S. rosmarinus* produced antiallodynic and antihyperalgesic effects, with numerically greater activity in females than in males in a 1:10 ratio. Experimental evidence suggests that non-polar metabolites of *S. rosmarinus* are involved in its efficacy, mediated in part by sex-dependent gonadal hormonal mechanisms for FM-type pain relief, such as ERβ receptors in females and androgens in males. One limitation was the lack of exploration of estrogen receptor involvement in the effect of the non-polar *S. rosmarinus* extract in males. Blocking estrogen synthesis to eliminate endogenous estrogen production using an aromatase inhibitor such as 4-hydroxyandrostenedione could be useful in determining selective estrogen receptor modulation, which, based on the current results, will be interesting to explore in future targeted experiments.

## Figures and Tables

**Figure 1:**
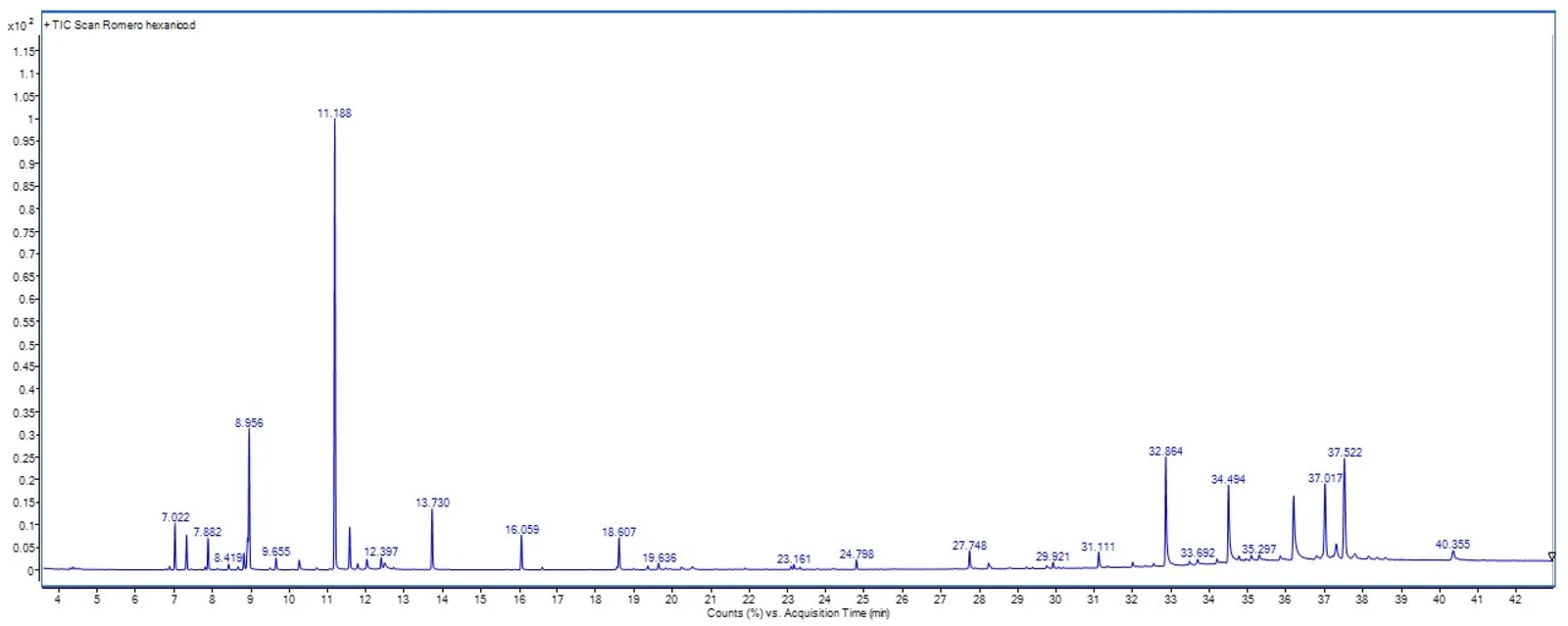
Total ion current chromatogram of the *S. rosmarinus* hexane extract.

**Figure 2:**
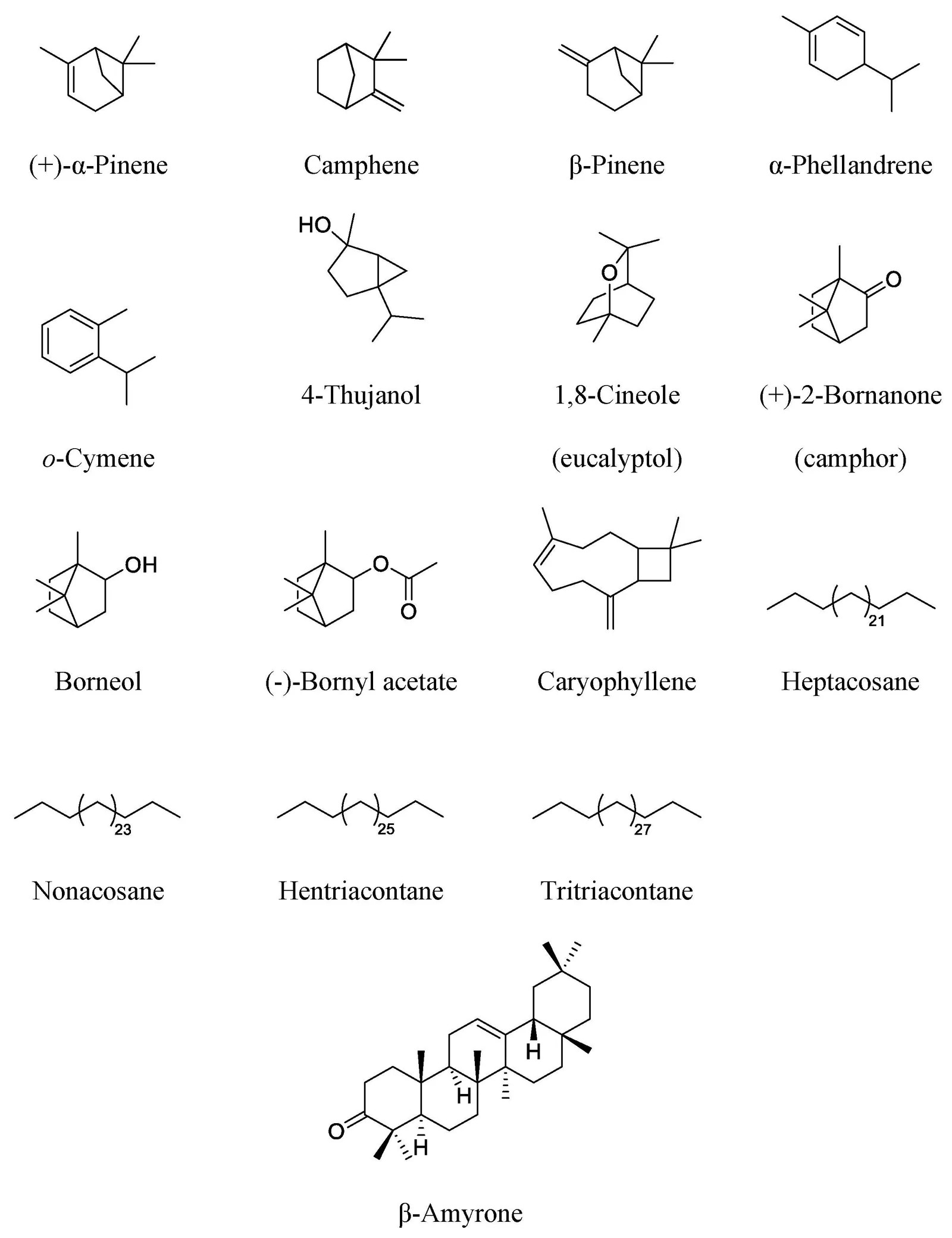
Chemical structure of the 16 compounds identified in the hexane extract of *S. rosmarinus*.

**Figure 3:**
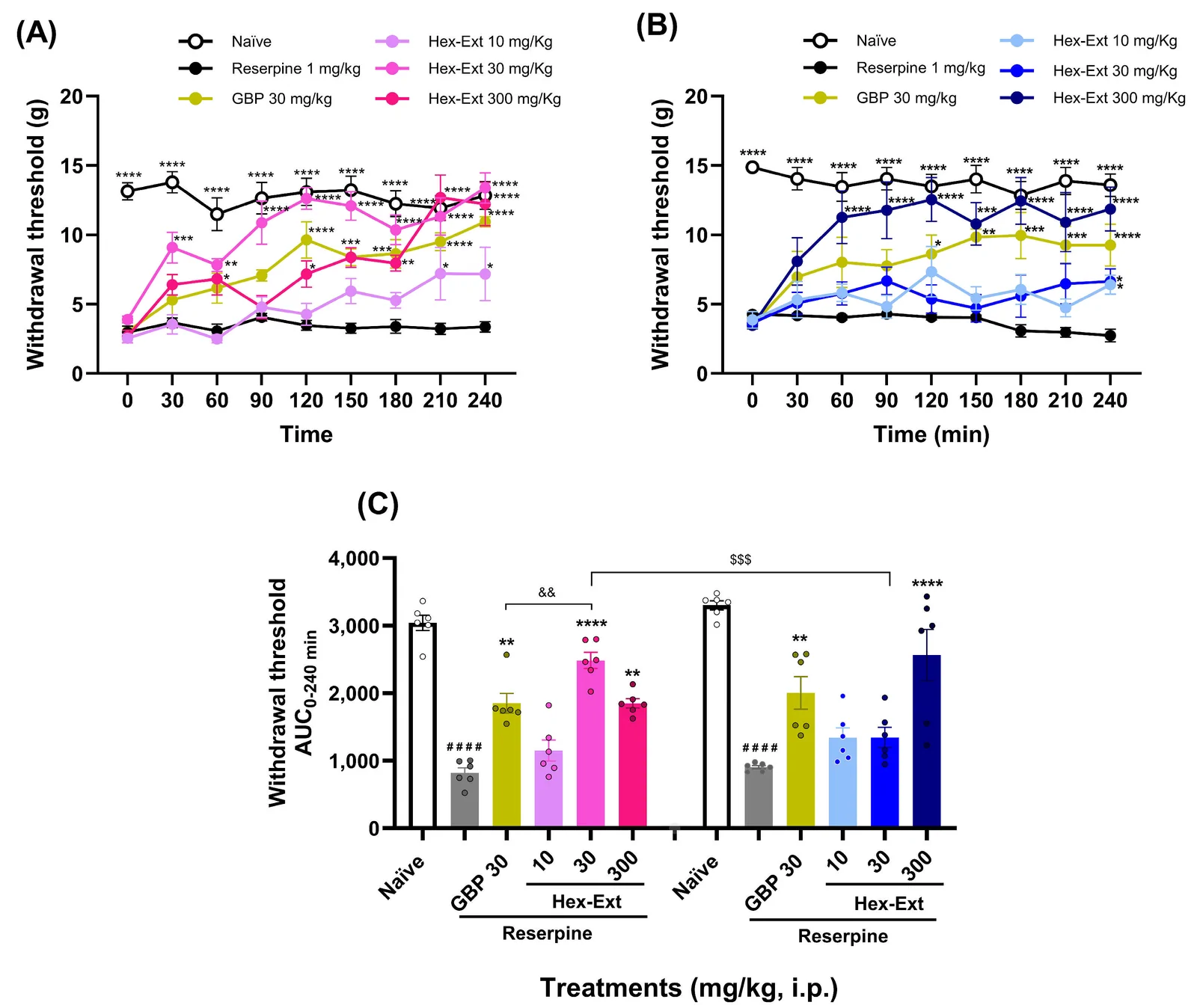
Time-course curves of the antiallodyne effect of *Salvia rosmarinus* extract (Hex-Ext) and gabapentin (GBP) of reserpinized (**A**) female and (**B**) male rats in tactile allodynia. Each point represents the mean ± SEM of n = 6 rats. Two-way ANOVA followed by Tukey’s post hoc test, * *p* < 0.05, ** *p* < 0.01, *** *p* < 0.001, and **** *p* < 0.0001 vs. the reserpine group (1 mg/kg, s.c.). (**C**) Area under the curve (AUC) obtained from the time courses of the antiallodynic effect observed in female and male rats. Each bar represents the mean ± SEM of 6 rats. Two-way ANOVA followed by Tukey’s post hoc test, statistical differences are represented as ^####^ *p* < 0.0001 reserpine vs. naïve group; ** *p* < 0.01 and **** *p* < 0.0001 Hex-Ext vs. the reserpine group; ^$$$^ *p* < 0.001 Hex-Ext (30 mg/kg) female rats vs. Hex-Ext (30 mg/kg) male rats. Student’s *t*-test, ^&&^ *p* < 0.01 Hex-Ext (30 mg/kg) vs. GBP (30 mg/kg).

**Figure 4:**
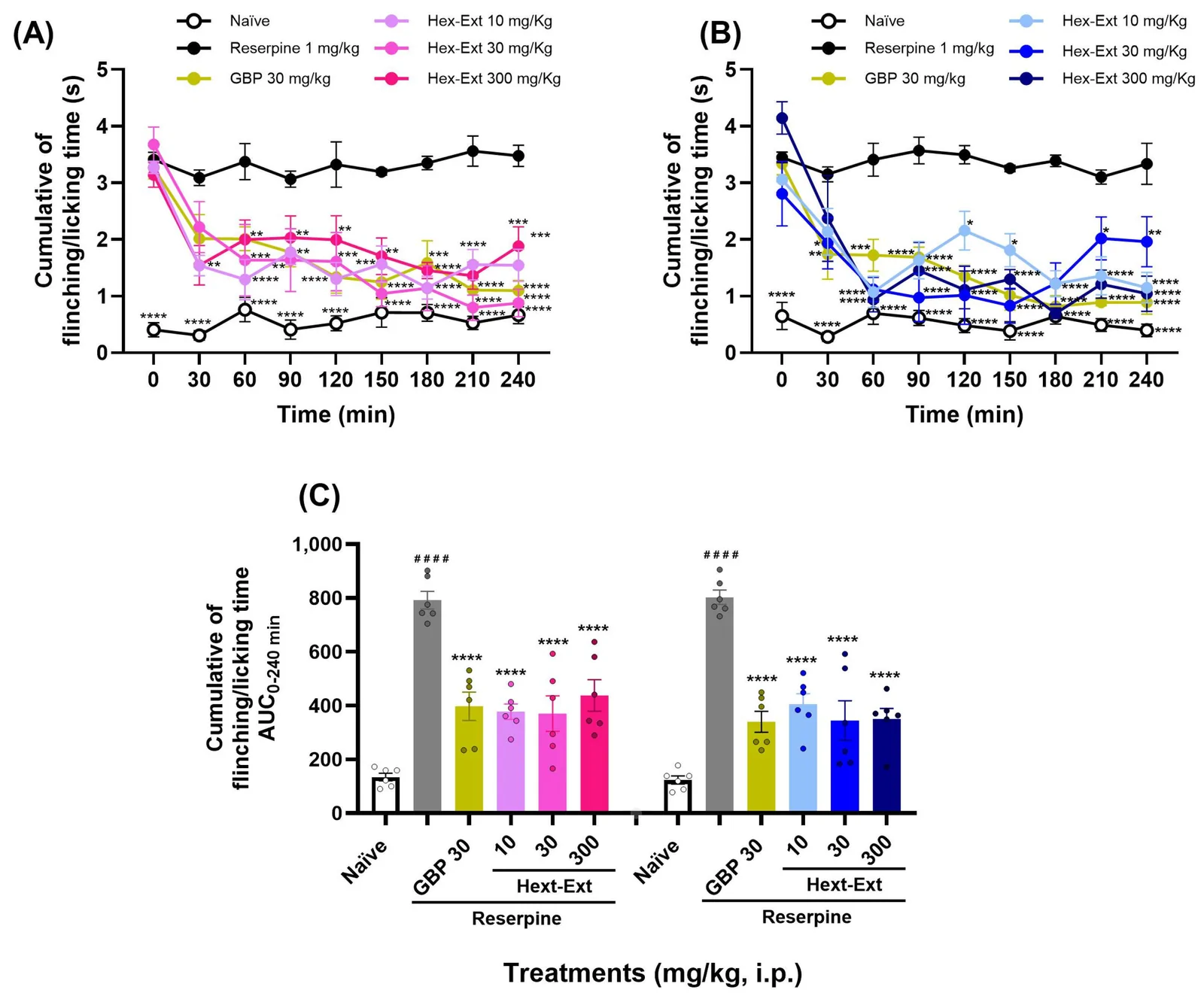
Time-course curves of the antiallodynic effect of *Salvia rosmarinus* extract (Hex-Ext) and gabapentin (GBP) in reserpinized (**A**) female and (**B**) male rats in thermal allodynia. Each point represents the mean ± SEM of n = 6 rats. Two-way ANOVA followed by Tukey’s post hoc test, * *p* < 0.05, ** *p* < 0.01, *** *p* < 0.001, and **** *p* < 0.0001 vs. the reserpine group (1 mg/kg, s.c.). (**C**) Area under the curve (AUC) obtained from the time courses of the antiallodynic effect obtained in female or male rats. Each bar represents the mean ± SEM of 6 rats. Two-way ANOVA followed by Tukey’s post hoc test. Statistical differences are represented as ^####^ *p* < 0.0001 reserpine vs. naïve group; **** *p* < 0.0001 Hex-Ext and GBP vs. the reserpine group.

**Figure 5:**
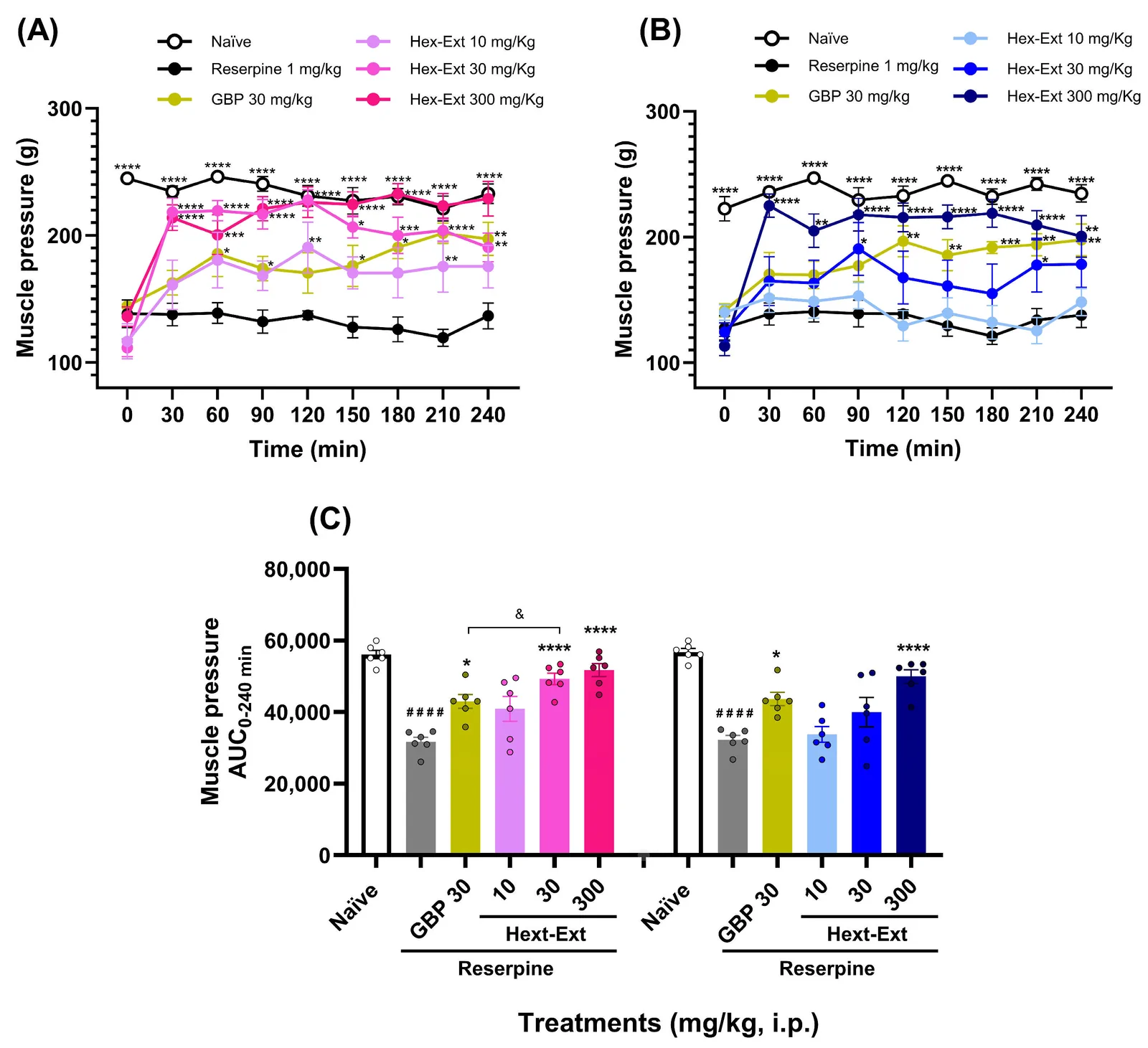
Time-course curves of the antihyperalgesic effect of the *Salvia rosmarinus* extract (Hex-Ext) and gabapentin (GBP) in reserpinized (**A**) female or (**B**) male rats in mechanical hyperalgesia. Each point represents the mean ± SEM of n = 6 rats. Two-way ANOVA followed by Tukey’s post hoc test, * *p* < 0.05, ** *p* < 0.01, *** *p* < 0.001, and **** *p* < 0.0001 vs. the reserpine group (1 mg/kg, s.c.). (**C**) Area under the curve (AUC) obtained from the time courses of the antihyperalgesic effect observed in female or male rats. Each bar represents the mean ± SEM of 6 rats. Two-way ANOVA followed by Tukey’s post hoc test. Statistical differences are represented as ^####^ *p* < 0.0001 reserpine vs. naïve group; * *p* < 0.05 and **** *p* < 0.0001 GBP or Hex-Ext vs. the reserpine group. Student’s *t*-test, ^&^ *p* < 0.05 Hex-Ext (30 mg/kg, females or 300 mg/kg, males) vs. GBP (30 mg/kg).

**Figure 6:**
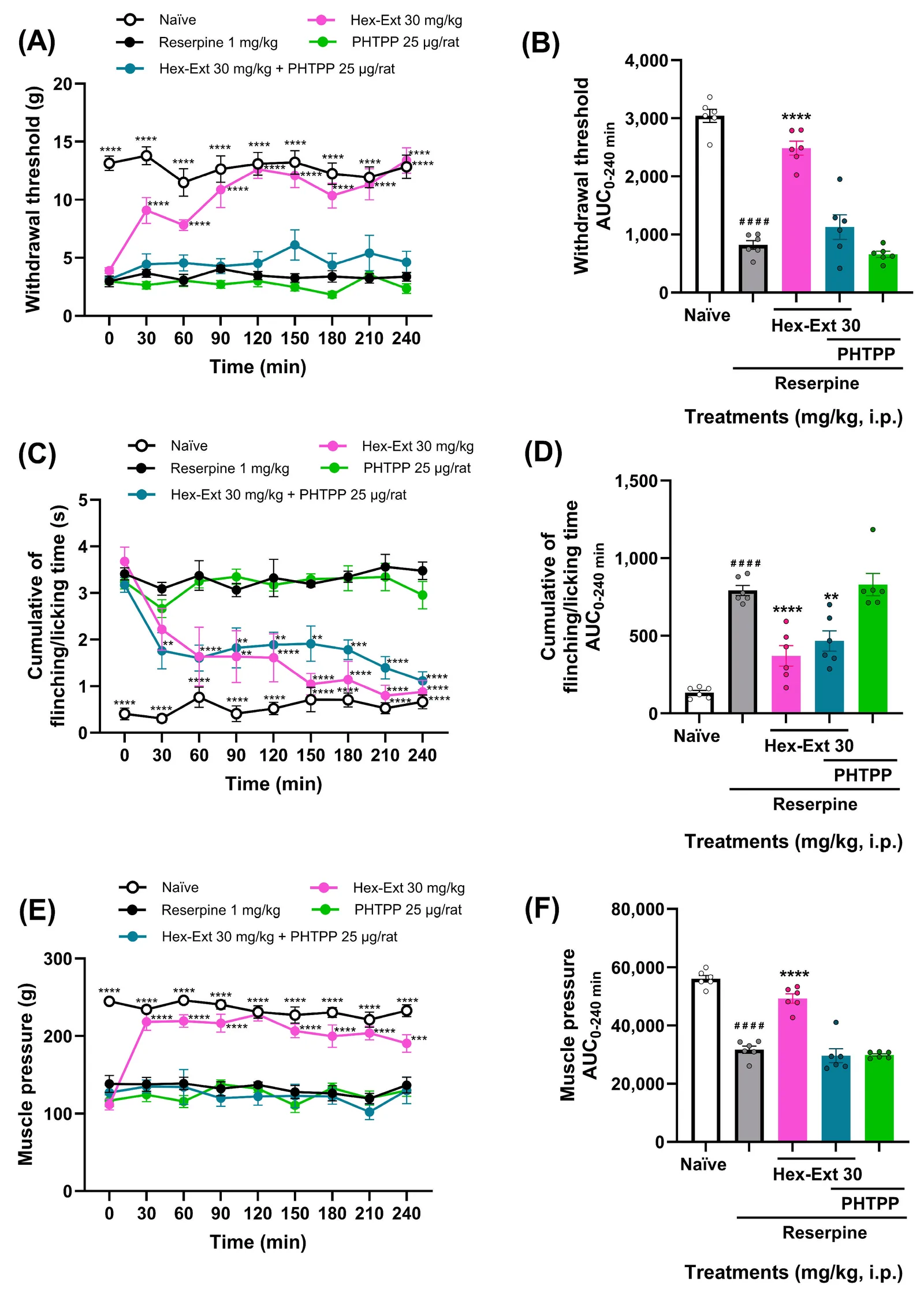
Time-course curves of the antiallodynic and antihyperalgesic effects of *Salvia rosmarinus* extract (Hex-Ext) (30 mg/kg, i.p.) in female rats alone and in the presence of PHTPP (25 µg/rat) in (**A**) tactile and (**C**) thermal allodynia, as well as in (**E**) mechanical hyperalgesia. Each point represents the mean ± SEM of n = 6 rats. Two-way ANOVA followed by Tukey’s post hoc test, ** *p* < 0.01, *** *p* < 0.001, and **** *p* < 0.0001 vs. the reserpine group (1 mg/kg, s.c.). Area under the curve (AUC) obtained from the time courses of the *S. rosmarinus* hexane extract (Hex-Ext) (30 mg/kg, i.p.), alone and combined with PHTPP (25 µg/rat) in (**B**) tactile and (**D**) thermal allodynia, as well as in (**F**) mechanical hyperalgesia. Each bar represents the mean ± SEM of n = 6 rats. One-way ANOVA followed by Tukey’s post hoc test. ^####^ *p* < 0.0001 reserpine vs. naïve groups; ** *p* < 0.01 and **** *p* < 0.0001 vs. reserpine.

**Figure 7:**
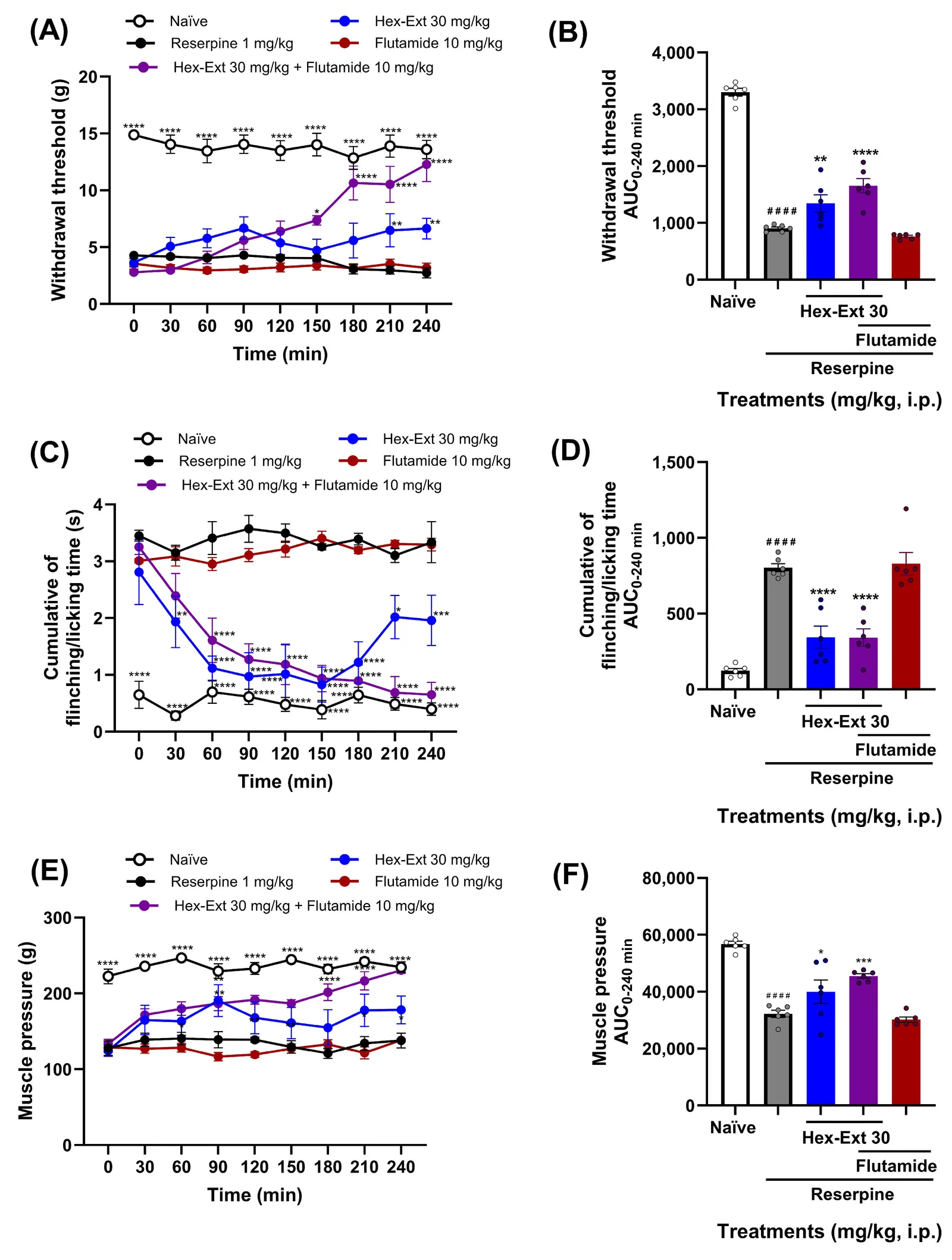
Time-course curves of the *Salvia rosmarinus* extract (Hex-Ext, 30 mg/kg, i.p.) effects on (**A**) tactile and (**C**) thermal allodynia and (**E**) hyperalgesia alone and in the presence of flutamide (10 mg/kg). Each point represents the mean ± SEM of n = 6 male rats. Two-way ANOVA followed by Tukey’s post hoc test, * *p* < 0.05, ** *p* < 0.01, *** *p* < 0.001 and **** *p* < 0.0001 vs. the reserpine group (1 mg/kg, s.c.). Area under the curve (AUC) obtained from the time courses of the effects of *S. rosmarinus* extract (Hex-Ext, 30 mg/kg, i.p.), flutamide (10 mg/kg, i.p.), and their combination on (**B**) tactile and (**D**) thermal antiallodynia and (**F**) mechanical hyperalgesia after their administration. Each bar represents the mean ± SEM of n = 6 rats. One-way ANOVA followed by Tukey’s post hoc test. ^####^ *p* < 0.0001 reserpine vs. naïve groups; * *p* < 0.05, ** *p* < 0.01, *** *p* < 0.001, and **** *p* < 0.0001 vs. reserpine.

**Figure 8:**
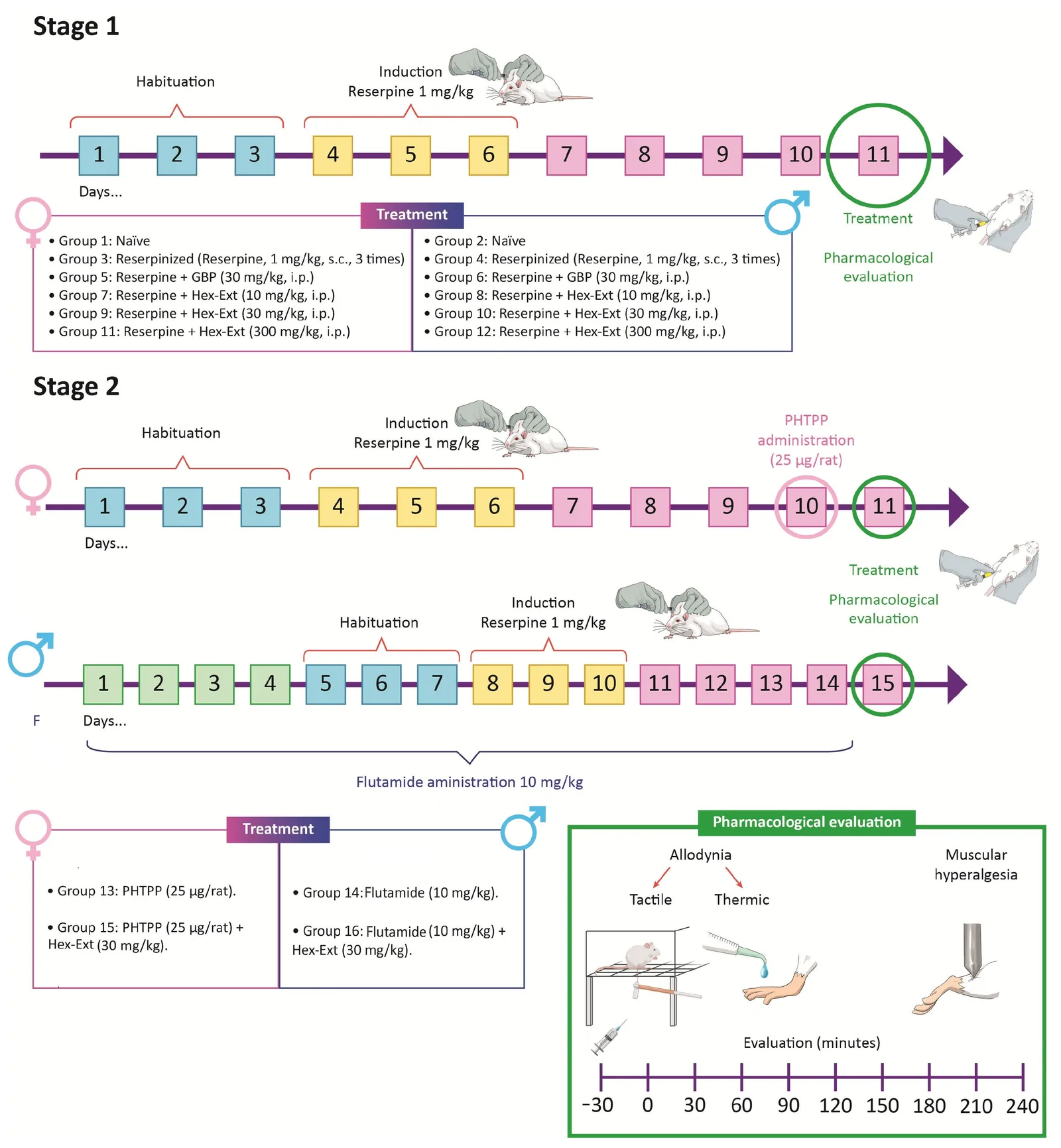
Timeline of the experimental design. **Stage 1:** Dose–response evaluation and **Stage 2:** hormonal mechanism of action during allodynia and hyperalgesia in experimental fibromyalgia in female rats (impairs groups) and male rats (pair groups).

**Table 1:** Identified compounds in the hexane extract of *S. rosmarinus*.

Peak	Rt (min)	%Relative Area	Compound	Molecular Formula	MW (g/mol)
1	7.022	2.23	(+)-α-Pinene	C_10_H_16_	136.24
2	7.326	1.71	Camphene	C_10_H_16_	136.24
3	7.882	1.66	β-Pinene	C_10_H_16_	136.24
4	8.419	0.33	α-Phellandrene	C_10_H_16_	136.24
5	8.82	0.92	*o*-Cymene	C_10_H_14_	134.22
6	8.917	0.48	4-Thujanol	C_10_H_18_O	154.25
7	8.956	5.34	1,8-Cineole (eucalyptol)	C_10_H_18_O	154.25
8	11.188	24.47	(+)-2-Bornanone (camphor)	C_10_H_16_O	152.24
9	11.576	2.37	Borneol	C_10_H_18_O	154.25
10	13.73	3.38	(-)-Bornyl acetate	C_12_H_20_O_2_	196.29
11	16.059	1.96	Caryophyllene	C_15_H_24_	204.36
12	31.111	1.07	Heptacosane	C_27_H_56_	380.75
13	32.864	8.15	Nonacosane	C_29_H_60_	408.80
14	34.494	5.65	Hentriacontane	C_31_H_64_	436.85
15	36.196	7.58	Tritriacontane	C_33_H_68_	464.91
16	37.017	6.47	β-Amyrone	C_30_H_48_O	424.71

## Data Availability

The presented data in this study are available upon request.

## Abbreviations

The following abbreviations are used in this manuscript:

ANOVA	Analysis of Variance
ATP	Adenosine Triphosphate
AUC	Area Under the Curve
CDMX	Ciudad de Mexico
CONAHCYT	Consejo Nacional de Humanidades, Ciencias y Tecnología
CNS	Central Nervous System
DMSO	Dimethyl Sulfoxide
ERβ	Estrogen Receptor β
FM	Fibromyalgia
GC-MS	Gas Chromatography coupled to Mass Spectrometry
GBP	Gabapentin
Hex-Ext	Hexane extract
i.p.	Intraperitoneal
INPRFM	Instituto Nacional de Psiquiatría Ramón de la Fuente Muñiz
LANCIC	Conservación del Patrimonio Cultural
MW	Molecular weight
NIST	National Institute of Standards and Technology
NMDA	N-Methyl-D-Aspartate
PHTPP	4-[2-phenyl-5,7-bis(trifluoromethyl)pyrazolo[1,5-a]pyrimidine-3-yl]phenol
RT	Retention time
s.c.	subcutaneous
SECIHTI	Secretaria de Ciencia, Humanidades, Tecnología
5-HT	Serotonin
5-HT_1A_	Serotonin Receptor Type 1A
TRP	Transient Receptor Potential
VMAT	Vesicular Monoamine Transporter
